# Predicting topical drug clearance from the skin

**DOI:** 10.1007/s13346-020-00864-8

**Published:** 2020-11-08

**Authors:** Maria Alice Maciel Tabosa, Magdalena Hoppel, Annette L. Bunge, Richard H. Guy, M. Begoña Delgado-Charro

**Affiliations:** 1 Department of Pharmacy & Pharmacology, University of Bath, Bath, UK; 2 Department of Chemical & Biological Engineering, Colorado School of Mines, CO, USA

**Keywords:** Skin, Dermato-pharmacokinetics, Skin clearance, Transdermal patch, Drug disposition

## Abstract

For topical drug products that target sites of action in the viable epidermal and/or upper dermal compartment of the skin, the local concentration profiles have proven difficult to quantify because drug clearance from the viable cutaneous tissue is not well characterised. Without such knowledge, of course, it is difficult—if not impossible—to predict a *priori* whether and over what time frame a topical formulation will permit an effective concentration of drug within the skin ‘compartment’ to be achieved. Here, we test the hypothesis that valuable information about drug disposition, and specifically its clearance, in this experimentally difficult-to-access compartment (at least, *in vivo*) can be derived from available systemic pharmacokinetic data for drugs administered via transdermal delivery systems. A multiple regression analysis was undertaken to determine the best-fit empirical correlation relating clearance from the skin to known or easily calculable drug properties. It was possible, in this way, to demonstrate a clear relationship between drug clearance from the skin and key physical chemical properties of the drug (molecular weight, log *P* and topological polar surface area). It was further demonstrated that values predicted by the model correlated well with those derived from *in vitro* skin experiments.

**Supplementary Information:**

The online version contains supplementary material available at 10.1007/s13346-020-00864-8.

## Introduction

Skin disease affects millions of people worldwide [1]. The effective delivery of a locally acting, dermatological drug demands knowledge of its ‘skin pharmacokinetics’ to determine the rate and extent with which it reaches its site of action in the epidermis/dermis. Of necessity, this requires understanding of not only the input rate of the drug into the skin but also its clearance from the ‘skin compartment’ into the systemic circulation. In this work, the phrase ‘clearance from the skin’ is used to mean the volume of skin from which a drug is completely removed per unit time.

For topical drug products that target sites of action in the viable epidermis and/or upper dermal compartment of the skin, the local concentration profiles have proven difficult to quantify because both drug input into the viable cutaneous tissue and its clearance therefrom are not well characterised [2]. Without such knowledge, of course, it is difficult—if not impossible—to predict a *priori* whether and over what time frame a topical formulation will permit an effective concentration of drug to be achieved within the skin compartment.

Mathematical and pharmacokinetic modelling has made a substantial contribution to the interpretation of drug movement and disposition in the skin [3, 4]. However, given the multistep nature of the dermal absorption process, the many formulation types and the complex nature of the physiological barrier, all models suffer from one or more limitations. For example, the need to ‘guesstimate’ several parameters to permit simulations to be performed, or the incorrect relation of ‘rate constants’ to drug physicochemical parameters, or the inability to obtain a prediction of drug concentration in the viable epidermis, means that many models (for most dermal products) are of little practical use.

There is a need, therefore, to develop simple, yet realistic and mechanistically meaningful, models to estimate the key dermato-pharmacokinetic parameters. Among the approaches currently under investigation are physiologically based pharmacokinetic (PBPK) models, which consist of physiologically realistic compartmental structures into which input parameters from different sources (e.g. *in silico* predictions, *in vitro* or *in vivo* experiments) can be combined to predict plasma and/or tissue concentration–time profiles [5, 6].

PBPK models take into account physiological properties of the tissue in question, which are not dependent on the drug and can, therefore, be applied to any compound, as well as characteristics intrinsic to the drug. PBPK models employ a ‘bottom-up’ approach, as opposed to the ‘top-down’ approach of classical pharmacokinetic models (e.g. one- or two-compartment approaches) [7]. That is, rather than estimating model parameters based on *in vivo* data (commonly derived from plasma/blood concentration versus time profiles), PBPK model parameters are determined a *priori* from *in vitro* experiments, *in silico* predictions or, if required, *in vivo* data.

Nonetheless, most PBPK models require a level of parameter calibration and/or optimisation. In general, a drug’s concentration in plasma is determined by the systemic volume of distribution at steady-state (*V*_SS_, L), defined as the total amount of drug in the body divided by the drug concentration in the plasma [8], and clearance (Cl, L/h), which is the volume of fluid (plasma or blood) cleared of drug per unit time. Assuming a simple one-compartment model with 1st-order elimination from the tissue compartment, the ratio of these independent physiological parameters provides the systemic elimination rate constant *k*_e_ (Eq. 1):

1 $${k}_{\mathrm{e}}=\mathrm{ Cl}/{V}_{\mathrm{SS}}$$

*V*_SS_ is an apparent volume that describes the extent of drug distribution and binding to the tissues and plasma (Eq. 2):

2 $${V}_{\mathrm{SS}}= {V}_{\mathrm{plasma}}+ \sum_{1}^{n}{K}_{\mathrm{tissue}/\mathrm{plasma}}\times {V}_{\mathrm{tissue},i}\times \left(1- {E}_{\mathrm{i}}\right)$$

where *V*_plasma_ is the volume of the plasma and *V*_tissue,*i*_ is the volume of the *i*th tissue; *K*_tissue/plasma_ is the tissue-to-plasma partition coefficient; *E*_i_ is the tissue extraction ratio and, for non-eliminating tissues, as is generally true of the skin [9]; and *E*_i_ equals 0.

**Fig. 1:**
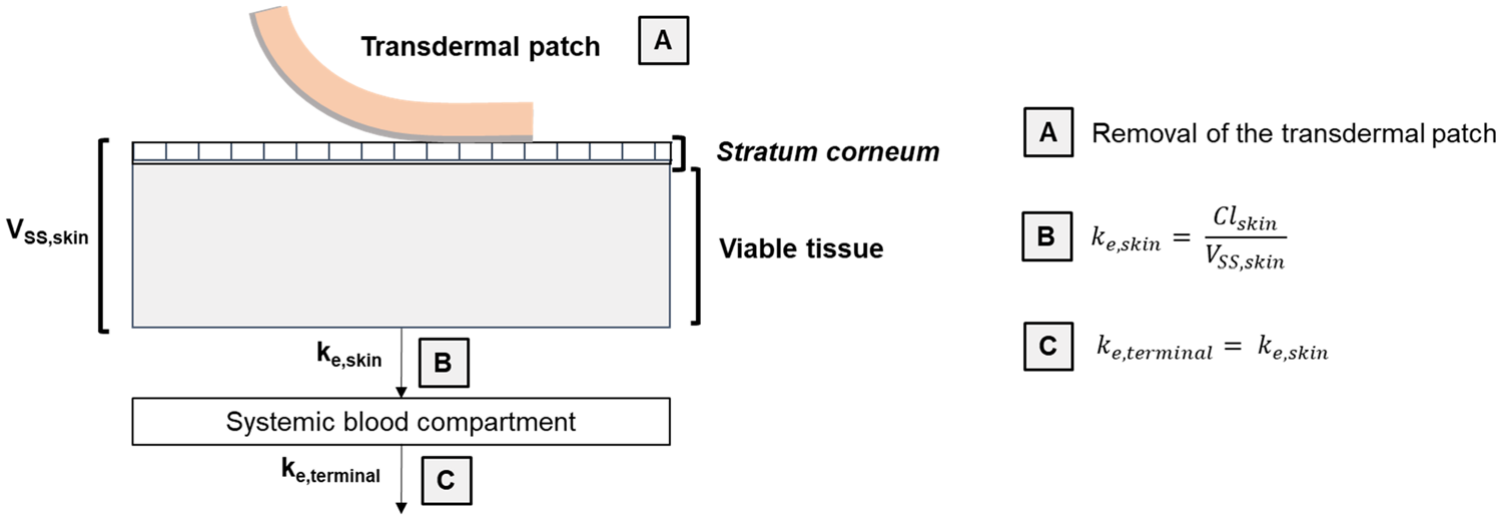
Schematic of the approach for estimating drug clearance from the skin from post-transdermal patch

In this paper, we test the hypothesis that valuable information about drug disposition, and specifically its clearance from the skin, can be derived from available systemic pharmacokinetic data for drugs administered via transdermal delivery systems. When a transdermal patch is applied, the drug delivery rate to the skin has been determined (the ‘input rate’) and the resulting systemic plasma concentration versus time profile has been measured both during patch wear and after its removal. The decline in the systemic plasma concentration post-patch removal is characterised by a terminal systemic rate constant (*k*_terminal_) that typically is much smaller than the elimination rate constant determined following intravenous administration, demonstrating clearly that ‘flip-flop’ kinetics are operative [10]. In other words, in transdermal drug delivery, the skin desorption rate is normally much slower than the systemic elimination. As a result, the disposition of a drug following transdermal application is usually rate-controlled by skin desorption. We have therefore assumed that the terminal rate constant post-patch removal reflects the elimination rate constant from the skin (i.e. *k*_e,skin_ = *k*_terminal_). In addition, we hypothesise as illustrated in Fig. 1 that, in flip-flop conditions, *k*_terminal_ is related to the drug’s clearance from the skin (Cl_skin_), via the corresponding ‘local’ volume of distribution (*V*_SS,skin_), and therefore, *k*_e,skin_ can be related to key physicochemical parameters of the drug.

To test this hypothesis, the transdermal delivery literature has been searched and values of *k*_terminal_ identified for the 18 drugs present in 25 FDA-approved products (FDA Orange Book database of the end of 2017) for this route of administration (see Table 1). Information was also included (Table 1) on a lidocaine patch, for which systemic pharmacokinetic data are available, even though this product is administered to elicit a local rather than systemic pharmacological effect. Importantly, the physicochemical properties of these transdermal drugs are quite broad: for example, molecular weights (MW) between 160 and 470 Da and logarithm of the octanol–water partition coefficient (log *P*) values from ~ 1.0 to 5.0 (Table 2).

**Table 1:** Reported values of *k*_terminal_, *V*_SS_/BW, *F*_i,pH=7.4_, *f*_u,p_ and predicted values for *K*_(skin/p)_ and *V*_SS,skin_/BW of 19 transdermal drugs

Drug	Trade name	*k*_e,terminal_^a^ (h^−1^)	*V*_SS_/BW^b^ (L kg^−1^)	*F*_i,pH=7.4_^c^	*f*_u,p_^d^	*K*_(skin/p)_^e^	*V*_SS,skin_/*A*_skin_^f^ (cm)
Buprenorphine	Butrans®	0.025	6.1	0.87	0.04	3.5	0.50
Clonidine	Catapress-TTS®	0.037	2.9	0.81	0.80	1.6	0.24
Estradiol^g^	Estraderm®	0.22	1.0	0	0.02	2.0	0.30
	Climara®						
	Vivelle®						
	Alora®						
	Vivelle-dot®						
	Menostar®						
	Minivelle®						
Estradiol (E) and norethisterone acetate^h^ (NAc)	Combipatch®	E: 0.22/NAc: 0.046	E: 1.0/NAc: 4.0	0	E: 0.02/NAc: 0.03	E: 2.0 / NAc: 4.2	E: 0.30/NAc: 0.57
Estradiol (E) and levonorgestrel (L)	Climara Pro®	E: 0.22/L: 0.021	E: 1.0/L: 1.8	0	E: 0.02/L: 0.06	E: 2.0/L: 2.3	E: 0.30/L: 0.34
Ethinyl estradiol (EE) and norelgestromin^j^ (N)	Xulane®	EE: 0.05/N: 0.025	EE: 5.0/N: 3.0	0	EE: 0.05/N: 0.03	EE: 4.4/N: 4.2	EE: 0.64/N: 0.62
Fentanyl	Duragesic®	0.034	6.0	0.97	0.16	3.2	0.46
Glyceryl trinitrate^j^	Nitro-Dur®	2.08	3.3	0	0.40	2.3	0.33
	Minitran®						
Granisetron	Sancuso®	0.021	3.0	1.00	0.35	1.7	0.25
Lidocaine	Lidoderrn®	0.12	1.5	0.78	0.38	1.1	0.16
Methylphenidate	Daytrana®	0.20	2.6	0.99	0.85	1.6	0.22
Nicotine	Nicoderm CQ®	0.21	2.5	0.75	0.95	1.4	0.21
	Habitrol®						
Oxybutynin	Oxytron®	0.061	2.8	0.86	0.09	1.9	0.28
Rivastigmine	Exelon®	0.25	2.2	0.94	0.60	1.4	0.21
Rotigotine	Neupro®	0.125	53.8	0.96	0.10	14.5	2.10
Scopolamine	Transderm Scop®	0.073	1.0	0.80	0.90	0.8	0.11
Selegiline	Emsam®	0.034	26.5	0.57	0.06	7.9	1.14
Testosterone	Androderm®	0.37	1.0	0	0.01	1.6	0.23

^a^Calculated from literature values of *t*_1/2_ assuming first-order terminal phase kinetics; sources are provided in Table S2 of the Supplementary Information

^b^Deduced from pharmacokinetic data obtained after administration of the drug; sources are provided in Table S1 of the Supplementary Information

^c^Calculated using Eq. 4 for the p*K*_a_ values listed in Table 2

^d^Extracted from [11]

^e^Calculated using correlations described in Table 3

^f^Calculated using Eq. 3

^g^Also reported as estradiol

^h^Also reported as norethindrone acetate

^i^Also reported as norelgestromin

^j^Also known as nitroglycerin(e)

**Table 2:** Physicochemical properties of the 19 drugs considered

Drug	Molecular descriptors										
	MW (Da)	MV (cm^3^ mol^−1^)	log *P*	log D_7.4_	MP (°C)	TPSA (Å^2^)	HBD	HBA	HBT	RotB	p*K*_a_
Buprenorphine	467.6	368.2	4.98	3.85	219	62.2	2	5	7	5	8.3/9.5
Clonidine	230.1	153.1	1.59	1.33	130	36.4	2	3	5	1	8.0
Estradiol	272.4	232.6	4.01	3.36	179	40.5	2	2	4	0	10.3
Ethinyl estradiol	296.4	244.4	3.67	3.54	163	40.5	2	2	4	1	10.3
Fentanyl	336.5	309.3	4.05	3.44	86	23.5	0	3	3	6	9
Glyceryl trinitrate	227.1	135.8	1.62	2.00	13	174.2	0	12	12	8	-
Granisetron	312.4	234.8	2.12	1.04	156	50.2	1	5	6	2	10.5/12.3
Levonorgestrel	312.4	274.3	3.33	3.15	228	37.3	1	2	3	2	13.6
Lidocaine	234.3	228.3	2.44	1.66	69	32.3	1	3	4	5	8
Methylphenidate	233.3	218.0	2.33	0.70	225	38.3	1	3	4	4	9.4
Nicotine	162.2	157.1	1.17	0.13	-79	16.1	0	2	2	1	3.2/8
Norelgestromin	327.5	265.0	4.34	4.07	226	52.8	2	3	5	2	11.3/13.1
Norethisterone acetate	340.5	296.1	3.72	3.55	171	43.4	0	3	3	3	-
Oxybutynin	357.5	325.7	3.96	3.83	130	49.8	1	4	5	10	8.2/12
Rivastigmine	250.3	241.1	2.24	2.30	67	32.8	0	4	4	5	8.6
Rotigotine	315.5	272.0	4.79	3.28	136	51.7	3	1	4	6	8.8/10.5
Scopolamine	303.1	230.9	0.98	0.51	59	71.5	1	5	6	5	8
Selegiline	187.3	196.1	2.90	2.75	142	3.2	0	1	1	5	7.5
Testosterone	288.4	256.9	3.32	3.02	138	37.3	1	2	3	0	-

*MW* molecular weight, *MV* molecular volume, *P* octanol–water partition coefficient, *D*_*7.4*_ octanol-pH 7.4 buffer distribution coefficient, *MP* melting point, *TPSA* topological polar surface area, *HBA* number of H-bond acceptors, *HBD* number of H-bond donors, *HBT* HBA + HBD, *RotB* number of rotatable bonds, *pK*_*a*_ negative log_10_(acid dissociation constant)

## Materials and methods

Evaluation of dermal drug clearance (Cl_skin_) involved the following steps: (i) identifying the terminal half-life (*t*_1/2_) and the corresponding terminal rate constant (*k*_terminal_) from the drug’s systemic plasma concentration versus time profile after removal of a transdermal patch, (ii) estimating the drug’s volume of distribution in the skin (*V*_SS,skin_) and, finally, (iii) calculating drug clearance from the skin assuming *k*_e,skin_ = *k*_terminal_.

### Identifying the terminal half-life (*t*_1/2_) and rate constant (*k*_terminal_)

A literature search of pharmacokinetic studies performed on 19 transdermally delivered drugs provided information from which the terminal half-lives (*t*_1/2_) were derived. The literature search used PubMed and different combinations of the keywords: ‘cutaneous’, ‘skin’, ‘transdermal patch’, ‘pharmacokinetic’ and ‘clearance’. Pharmacokinetic information was also obtained from relevant Drug@FDA public repositories (https://www.accessdata.fda.gov/scripts/cder/daf/, FDA-Clinical Pharmacology and Biopharmaceutics Review(s),). The data used were from healthy adults (18–71 years; *n* ≥ 5), and the *C*_p_ versus time profiles analysed had at least 3 measurements after patch removal. There were, however, two exceptions: (i) as almost all studies involving testosterone were performed on patients with hypogonadism, these data were accepted for analysis; and (ii) similarly, for methylphenidate, the only data available were from children (6–12 years).

When not specifically reported, *t*_1/2_ values were extracted from *C*_p_ versus time profiles assuming that the terminal phase kinetics were first-order (i.e. *t*_1/2_ = ln(2)/*k*_terminal_). The half-lives were derived from graphs using WebPlotDigitizer software (version 3.10, Ankit Rohatgi; Austin, TX, USA), and *k*_terminal_ values were deduced (Table 1 and Table S2) (together with the corresponding references) in the Supplementary Information). If data from more than one source were available, an arithmetic mean of the *k*_terminal_ values was calculated.

### Estimating the drug’s volume of distribution in the skin

Equation 2 indicates that each tissue/organ contributes to the overall total volume of distribution (*V*_SS_) of the drug and suggests that *V*_SS,skin_ can therefore be approximated by the following:

3 $${V}_{\mathrm{SS},\mathrm{skin}}/{A}_{\mathrm{skin}}= {(V}_{\mathrm{skin}}/{A}_{\mathrm{skin}})\times {K}_{(\mathrm{skin}/\mathrm{p})}$$

where *V*_skin_ and *A*_skin_ are respectively the volume and area of the skin compartment, and *K*_(skin/p)_ is the drug’s partition coefficient between the skin and the plasma. It is relevant to point out that protein binding can occur within the skin (in the stratum corneum (SC) and/or the viable skin) and that *K*_(skin/p)_ is likely to be greater than 1, therefore. Also, as most transdermal drugs are either neutral compounds or weak bases, the degree of ionisation of the latter is an important parameter to consider as well.

Correlations listed in Table 3 have been developed by Yun and Edginton [8] to predict *K*_(skin/p)_ in the rat from physicochemical descriptors (log *P*, degree of ionization (*F*_i_) and plasma protein binding (*f*_u,p_)) together with organism-specific information (specifically, the ratio of the systemic volume to body weight (*V*_SS_/BW, L kg^−1^) in the rat) for moderate to strong bases (p*K*_a_ ≥ 7.4, equation A) and for acids and neutral compounds, zwitterions and weak bases (p*K*_a_ ≤ 7.4, equation B) [8].

**Table 3:** Correlations for predicting drug skin-to-plasma partition coefficients (*K*_(skin/p)_) in rats. Table adapted from Yun and Edginton [8]

Equation	Regression equation	Number	*R*^2^
A	log *K*_(skin/p)_ = − 0.144 + 0.663(log*V*_SS_/BW) + 0.033(log *P*)	28	0.80
B	log *K*_(skin/p)_ = − 0.331 + 0.544(log*V*_SS_/BW) + 0.158(log *P*) − 0.318(*F*_i_) + 0.384(*f*_u,p_)	26	0.73

To adapt this approach to estimate *V*_SS,skin_ in humans using Eq. 3, *V*_skin_ and *A*_skin_ were assumed to be 2.6 L and 1.8 m^2^ (based on a standard body weight of 70 kg, [12]) and *K*_(skin/p)_ was determined from either equation A or B (Table 3) using the appropriate *V*_SS_/BW. We note that the dermis makes up 95–96% of the total skin weight in humans and the epidermis makes up the remainder [12]. As Yun and Edginton [8] did not distinguish between the different skin layers, it was assumed here that *K*_(skin/p)_ is the average value for all skin layers.

Ideally, in the selection of an appropriate model for determination of *K*_(skin/p)_, a determination must be made as to how accurately the selected model reflects the properties of the skin and the plasma. One needs to bear in mind that the correlations developed by Yun and Edginton [8] were built using *V*_SS_/BW from rats. In contrast, in this work, *K*_(skin/p)_ predictions were made using *V*_SS_/BW from humans. This approach was taken due to the absence, at least to the authors’ knowledge, of a model/correlation for *K*_(skin/p)_ prediction using human data. Although rat skin is commonly used in *in vitro* and *in vivo* percutaneous studies [13, 14], its properties do not perfectly mimic human skin [14–17]. However, in comparison with human skin, rat skin does have a similar *stratum corneum* (SC) thickness, although a slightly thinner epidermis and total skin thickness [14].

Values of the steady-state systemic drug volume of distribution per BW in humans (*V*_SS_/BW) and the plasma protein binding (*f*_u,p_) were obtained from the literature (Table 1). The relevant drug physicochemical properties are listed in Table 2. The degree of ionization (*F*_i_) at physiological pH 7.4 (*F*_i,pH=7.4_) for a chemical was calculated in the normal way using Eq. 4:

4 $${F}_{\mathrm{i},\mathrm{pH}=7.4}=1- \left(\frac{1}{1+ {10}^{g(7.4-\mathrm{p}{K}_{\mathrm{a}})}}\right)$$

where *g* = + 1 for a monoprotic acid and − 1 for monoprotic base. Table 1 lists *V*_SS,skin_/*A*_skin_ as well as the parameter values used in its calculation (i.e. *V*_SS_/BW, *f*_u,p_, *F*_i,pH = 7.4_ and *K*_(skin/p)_).

### Calculating drug clearance from the skin

Finally, assuming that skin pharmacokinetics can be described using a one-compartmental model with first-order elimination kinetics, Cl_skin_/*A*_skin_ (which has units of cm h^−1^, i.e. the same as those for the skin permeability coefficient) was calculated using Eq. 5.

5 $${\mathrm{Cl}}_{\mathrm{skin}}/{A}_{\mathrm{skin}}= {k}_{\mathrm{e},\mathrm{skin}} \times {V}_{\mathrm{SS},\mathrm{skin}}/{A}_{\mathrm{skin}}$$

### Drug physicochemical parameters

The molecular descriptors are as follows: log *P*, molecular weight (MW) and melting point (MP) are from EPA’s CompTox Chemistry Dashboard (https://comptox.epa.gov/dashboard), which includes experimental values that were used when available. When several experimental values were available, the mean value was used. In addition, the logarithm of the octanol–water distribution coefficient at pH = 7.4 (log D_7.4_), the number of rotatable bonds (RotB), the numbers of hydrogen-bond acceptors (HBA) and donors (HBD) and their sum (HBT), molecular volume (MV), topological polar surface area (TPSA) and p*K*_a_ were calculated using ACD/Labs (Toronto, Canada, version 5.0). The physicochemical properties of the 19 drugs are listed in Table 2.

### Multiple linear regression model development

Multiple linear regression (MLR), using the ordinary least squares (OLS) method, was used to develop an empirical relationship that best described the dependence of the log transformed area normalised skin clearance (log Cl_skin_/*A*_skin_) on the key physicochemical properties of the drug. Stepwise MLR was performed using The Unscrambler® X software (Version 10.5, Camo A/S, Oslo, Norway). In each regression analysis, a variable was either added or removed until the fit obtained had the highest adjusted and predicted coefficient of determination (*R*^2^) and when all the predictors were statistically significant (*p* value ≤ 0.05). In addition, collinearity between predictor values was assessed by screening the variance inflation factor (VIF) for each equation; a VIF = 5 was the cut-off criterion [18].

Subsequently, internal validations were undertaken to estimate the predictive value of the final model, defined by the determination coefficients of leave-25%-out (*Q*^2^_25%_) and of leave-one-out cross-validation (*Q*^2^_LOO_) [19]. In leave-25%-out cross-validation, the data set for the 19 compounds were randomly divided into training (*n* = 14) and test (*n* = 5) groups. Then, coefficients of determination (*R*^2^) and prediction (*Q*^2^_25%_) were obtained by regressing the parameters of the model equation to random combinations of the 14 training observations. The calculation of *Q*^2^_LOO_ involved the omission of the data for one drug and re-determining the regression model using the remaining 18 data. The resulting equation is then used to predict the dermal clearance of the omitted chemical. The correlation between the predicted and observed values in the newly generated dermal clearance data set is used to judge the fit. *Q*^2^_LOO_ is therefore able to validate the model without additional compounds or splitting the data.

### Model verification using *in vitro* skin permeation

*In vitro* permeation experiments were performed to measure the rate at which three drugs (buprenorphine (BUP), nicotine (NIC) and diclofenac (DF)) are cleared from the skin following application of examples of commercially available transdermal patches. The transdermal patches tested were the following: Transtec® (35 μg h^−1^, 20 mg of buprenorphine over 25 cm^2^) from NAPP (Cambridge, UK), Nicotinell® (7 mg/24 h, 17.5 mg of nicotine over 10 cm^2^) from Novartis (Camberley, UK) and Voltaren® (medicated plaster, 180 mg of diclofenac epolamine over 140 cm^2^) was from GlaxoSmithKline (Munich, Germany). Experiments were carried out in static, Franz diffusion cells (Permegear, Hellertown, PA, USA) with a receptor volume of ∼ 7.4 mL. Porcine skin (thickness ~ 750 µm) from a single pig, sourced, stored and prepared as previously described [20] was used. A 1.54-cm^2^ disk of the patch was applied to the skin surface before mounting in the Franz cell; 10 passes of a custom-made, weighted roller ensured complete adhesion between patch and skin. After assembly of the diffusion cell, the lower compartment was filled with a drug-specific receptor solution (Table 4). The patch was applied for a specific ‘uptake time’ and then removed; subsequently, the skin remained mounted in the diffusion cell for a further ‘clearance time’ (or times) (Table 4), at the end of which the experiment was terminated. Six replicates of all measurements were made. ‘Uptake times’ were chosen to be sufficiently long that steady-state diffusion had been achieved; ‘clearance times’ were selected such that an obviously significant reduction in the quantity of drug taken up into the skin had occurred without compromising the ability to detect that remaining.

**Table 4:** *In vitro* skin permeation experimental details

	Buprenorphine	Nicotine	Diclofenac
Receptor solution	20:80 PEG 400–PBS 10 mM + 0.01% sodium azide	PBS 10 mM	PBS 10 mM
‘Uptake’ time (h)	72	2	6
‘Clearance’ time(s) (h)	24	1.5, 3.0	5, 17, 24
Extraction solution^a^	40:60 ACN:TFA^a^	40:60 ACN–PBS 10 mM^b^	MeOH^a^

*PEG* polyethylene glycol, *PBS* phosphate-buffered saline (pH 7.4), *ACN* acetonitrile, *TFA* trifluoroacetic acid 0.03% *v*/*v*, *MeOH* methanol

^a^Extraction volumes were 1.5 mL for tape strips and either 4 mL (buprenorphine, diclofenac) or 8 mL (nicotine) for remaining skin. Mean extraction efficiencies from tape strips and from the remaining skin were > 89%.

After dismantling the diffusion cell, the SC was removed by repeated tape-stripping. Templates (Scotch® Book Tape, 3M, St. Paul, MN, USA), with a circular internal area that matched the 1.54-cm^2^ patch area, were positioned on the skin, and then a total of 20 adhesive tape strips (2.0 cm × 2.5 cm, Scotch® Book Tape) were sequentially applied, pressed down firmly and quickly removed. Drug was extracted from the individual tapes (Table 4) and the total amount therein was quantified by HPLC (Dionex, UK) (Table 5). The quantity of drug in the remaining skin tissue was also determined with the same analytical procedure after extraction. The concentrations of the three drugs in the receptor solution (measured at the end of each experiment by HPLC) were always less than one-tenth of the corresponding aqueous solubilities confirming that sink conditions were maintained. HPLC assays with UV detection, running Chromeleon software, were developed for the three drugs. A HiQSil C18HS analytical reverse phase column (150 × 4.6 mm i.d.; 5 µm particle size) (Kromatek, UK) was used. The chromatographic conditions are provided in Table 5.

**Table 5:** HPLC–UV method conditions used for quantification of BUP, NIC and DF

HPLC parameters	Buprenorphine	Nicotine	Diclofenac
Mobile phase^a^	22:19:59 ACN:MeOH:TFA	35:35:30 ACN:MeOH:PBS	75:25 MeOH:formic acid^b^
Oven temperature (°C)	25	25	40
Flow rate (mL min^−1^)	1.0	1.0	1.2
Retention time (min)	11.8	3.2	7.3
Injection volume (µL)	75	50	100
UV detection (nm)	220	260	280
Limit of quantification (µg mL^−1^)	0.14	0.12	0.10

^a^All abbreviations as defined in Table 4

^b^0.1% *v*/*v* in water

## Results and discussion

**Fig. 2:**
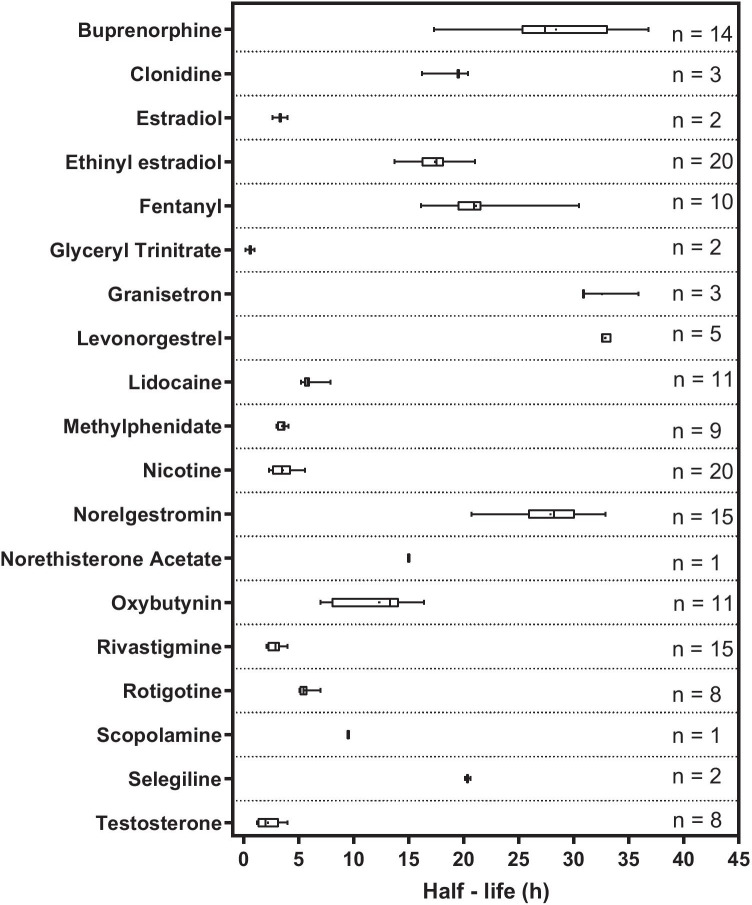
Box-and-whisker plot of the systemic terminal half-life (*t*_1/2_) values reported in the literature for 19 drugs following transdermal patch removal. The boxes comprise the median (line), mean (dot) and 25th and 75th percentile (ends) values. The bars reflect the minimum and maximum values observed. The number (*n*) of half-lives found for each drug are listed on the right of the graph

The analysis of the literature for 19 different drugs identified more than 70 specific studies that yielded a total of 160 terminal half-life values. In some cases (such as scopolamine and norethisterone acetate), only a single half-life was found while, for other drugs (such as nicotine and ethinyl estradiol), 20 individual half-life values were discovered. The distribution of the half-lives for each drug is presented as a box-and-whisker plot in Fig. 2. From the half-life value, *k*_terminal_ (= (ln 2)/*t*_1/2_) for each drug was then calculated (see ‘Supplementary Information’, Table S2 together with the corresponding references).

To estimate the area-normalised volumes of distribution in the skin (*V*_SS,skin_/*A*_skin_) of the 19 drugs, the skin-to-plasma partition coefficients (*K*_(skin/p)_) were first determined using the equations in Table 3; values ranged from 0.8 for scopolamine to 14.5 for rotigotine (Table 1). The relatively high *K*_(skin/p)_ of the latter drug was not unexpected as its high lipophilicity (log *P* = 4.7) and substantial systemic volume of distribution (*V*_SS_/BW = 53.8 L kg^−1^) already suggest that rotigotine is likely to accumulate in tissues. Likewise, scopolamine’s smaller lipophilicity (log *P* ~ 1) and relatively small volume of distribution (*V*_SS_/BW = 1.0 L kg^−1^) are completely consistent with its much smaller *K*_(skin/p)_. It is worth mentioning that there is only an 18-fold difference in skin partitioning between rotigotine and scopolamine despite the more than 4000-fold difference in their octanol–water partition coefficients. This is consistent with a skin compartment that is dominated by the ‘viable’ skin layers rather than the SC. The skin-plasma partitioning represents, therefore, the equilibrium between the relatively hydrophilic layers of the skin and hydrophilic plasma.

From the calculated *K*_(skin/p)_ for each drug and the *V*_skin_/*A*_skin_, *V*_SS,skin_/*A*_skin_ was then calculated from Eq. 3; the results are listed in Table 1. Finally, drug clearance from the skin (Cl_skin_/*A*_skin_ in cm h^−1^) was assessed using Eq. 5 and the resulting values are in Table 6.

**Table 6:** Cl_skin_/*A*_skin_ (expressed as a logarithm) determined via Eq. 5 from the experimental data and the corresponding values predicted from multiple linear regression (Eq. 6)

ID	Drug	log [Cl_skin_/*A*_skin_ (cm h^−1^)]	
		Experimental	Predicted
1	Buprenorphine	− 1.90	− 2.03
2	Clonidine	− 2.05	− 1.74
3	Estradiol	− 1.18	− 1.09
4	Ethinyl estradiol	− 1.59	− 1.41
5	Fentanyl	− 1.81	− 1.78
6	Glyceryl trinitrate	− 0.16	− 0.14
7	Granisetron	− 2.28	− 2.03
8	Levonorgestrel	− 2.15	− 1.71
9	Lidocaine	− 1.72	− 1.49
10	Methylphenidate	− 1.36	− 1.45
11	Nicotine	− 1.35	− 1.59
12	Norelgestromin	− 1.81	− 1.26
13	Norethisterone acetate	− 1.58	− 1.71
14	Oxybutynin	− 1.77	− 1.69
15	Rivastigmine	− 1.28	− 1.69
16	Rotigotine	− 0.58	− 1.00
17	Scopolamine	− 2.10	− 2.16
18	Selegiline	− 1.41	− 1.28
19	Testosterone	− 1.07	− 1.52

An empirical model was then derived, using multiple linear regression (MLR), to predict dermal drug clearance. MLR is a statistical technique that can use a number of molecular descriptors to identify predictive, albeit empirical, relationships in data sets. The advantage of MLR is its simplicity and the easily interpretable mathematical results. The sign of the coefficient derived for each molecular descriptor indicates whether it contributes positively or negatively to the predicted parameter and its magnitude is a measure of the relative importance. However, MLR works best when (i) the structure–activity relationship is linear, (ii) the set of molecular descriptors is independent (i.e. descriptors do not show collinearity) and (iii) the number of compounds in the training set exceeds the number of molecular descriptors by at least a factor of five [22].

**Fig. 3:**
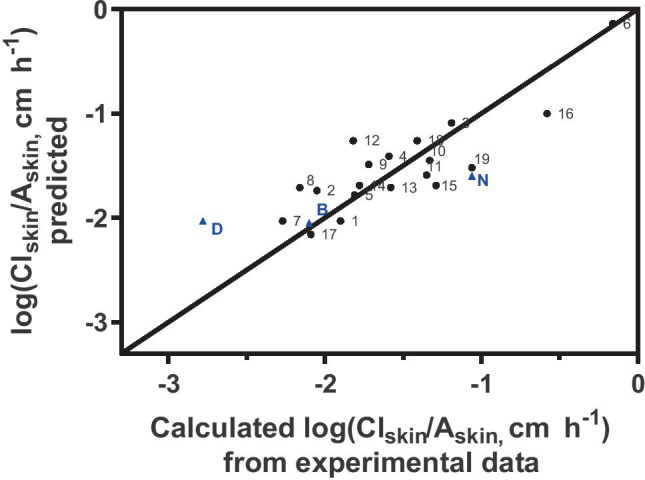
Relationship between log Cl_skin_/*A*_skin_ calculated from experimental data and that predicted by the MLR-derived Eq. 6. The solid line is the line of identity. The number against each point corresponds to that assigned to each of the 19 drugs in Table 6. The blue triangles show the predicted log Cl_skin_/*A*_skin_ values compared with those calculated from *in vitro* experiments (see Table 9 below) for buprenorphine (B), nicotine (N) and diclofenac (D)

At the outset, MLR was performed using ten molecular descriptors (MW, log *P*, MP, logD_7.4_, RotB, HBA, HBD, HBT, MV and TPSA) as potential predictors of drug clearance from skin; these values for the 19 drugs are listed in Table 2. In the end, a model based only on MW, log *P* and TPSA (in units of Å^2^) best explained the calculated dermal clearance (Eq. 6; Table 7; Fig. 3):

**Table 7:** Statistics of the descriptors of the MLR model (Eq. 6) developed to predict log(Cl_skin_/*A*_skin_)

Descriptor	Variance inflation value (VIF)	Derived coefficients ± standard error	*p* value
Intercept	–	− 0.921 ± 0.318	0.0110
MW	2.2	− 0.008 ± 0.002	0.0001
log *P*	2.2	0.389 ± 0.090	0.0006
TPSA	1.2	0.011 ± 0.002	0.0002

6 $${\mathrm{log}[\mathrm{Cl}}_{\mathrm{skin}}/\mathrm{Area }(\mathrm{cm }{\mathrm{h}}^{-1})]=-0.937-0.008\left(\mathrm{MW}\right)+0.391\left(\mathrm{log}P\right)+0.011(\mathrm{TPSA})$$

with an adjusted *R*^2^ = 0.67, *Q*^2^_LOO_ = 0.61 and *p* < 0.05 for all three variables; adjusted *R*^2^ is the square of the determination coefficient adjusted for degrees of freedom; *Q*^2^_LOO_ is the cross-validated (leave-one-out) square of the determination coefficient; and the *p* value is related to the significance of the parameters (Table 7).

The general principle of cross-validation is to split data into training and test sets. The former is used to fit the model while the latter serves to evaluate the fitted model’s predictive adequacy. Leave-one-out (LOO) cross-validation repeatedly partitions the data set into a training set which consists of all data points except one and then evaluates the predictive density for the held-out data point where predictions are generated based on the leave-one-out posterior distribution. The LOO estimator is nearly unbiased. Moreover, there was no evidence of collinearity in the predictors, with all VIF values being less than 5.

On the other hand, due to the small size of the data set here, the leave-25%-out cross-validation, which is normally employed when the data set is large, is highly variable and depends on which observations are in the training and test sets. To illustrate this point, the leave-25%-out cross-validation was performed 10 times and yielded the results in Table 8. Clearly, regressions using the same data set (but different subsets) produced quite different results (e.g. compare regressions II and X). Therefore, for the relatively small data set involved in this study, the LOO is a preferable and more appropriate method of validation.

**Table 8:** Coefficients of determination (*R*^2^) and determination coefficient of leave-25%-out (*Q*^2^25%) for 10 regressions on the same data set

Regression number	Drugs omitted (see Table 6 for code)	*R*^2^	*Q*^2^_25%_
I	1, 3, 9, 15, 19	0.81	0.25
II	7, 14, 15, 16, 18	0.73	0.81
III	8, 9, 10, 13, 19	0.79	0.09
IV	3, 5, 10, 13, 15	0.75	0.81
V	1, 7, 8, 9, 14	0.71	0.50
VI	5, 6, 13, 15, 16	0.50	0.81
VII	1, 12, 14, 17, 19	0.81	0.50
VIII	2, 8, 11, 14, 17	0.72	0.50
IX	3, 5, 7, 12, 19	0.79	0.25
X	6, 7, 9, 16, 18	0.40	0.36

In general, drug permeability across biomembranes, including the skin, increases with increasing permeant lipophilicity and decreases with increasing molecular size [23–26]. It is, therefore perhaps, not surprising that both log *P* and MW appear in the empirical relation describing drug clearance from the skin. TPSA was also found to be a significant predictor of drug clearance from the skin. TPSA is a molecular property related to the polarity, hydrogen-bonding potential and water solubility of organic molecules [27, 28]. TPSA has been shown to be inversely correlated with drug transport across the brain–blood barrier [29, 30] and the intestinal membrane [31–33], and the positive correlation found here is therefore somewhat surprising. While an increase in hydrogen bonding activity (both acceptor and donor) has been shown to result in a decrease in the partitioning into the organic phase due to the free energy cost associated with the disruption of the hydrogen bonds in the aqueous phase [34], the positive correlation observed with skin clearance may indicate the eventual importance of aqueous solubility in the sequential processes involved in drug elimination from the skin [35, 36]. Further speculation on this issue, in the absence of additional data, however, is not warranted at this time.

**Fig. 4:**
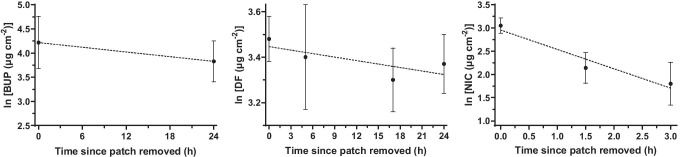
Amount of drug in the SC + epidermis/dermis (mean ± standard deviation; *n* = 6) as a function of the time of clearance. The slopes of the linear regressions (dashed lines) provide the drug elimination rate constants from the skin (*k*_e,skin_)

In an attempt to further validate the predictions of the model, a series of *in vitro* experiments were performed with three transdermal drug products. The amounts (A, normalised by patch area) of NIC, BUP and DF in the SC (removed by tape stripping), and in the remaining skin, were measured (a) immediately after patch removal (uptake) and, separately, (b) after further periods of time subsequent to patch removal (clearance) and used to calculate the elimination rate constant of the drugs from the skin (*k*_e,skin_). Assuming first-order kinetics, *k*_e,skin_ was estimated from the slope of the log-transformed mass (in the SC plus in the epidermis/dermis) versus time data of each drug (Fig. 4). The human systemic steady-state volumes of distribution (*V*_SS_/BW) of the drugs were from the literature, and the human skin-to-plasma partition coefficients (*K*_(skin/p)_) were estimated using equation A in Table 3. The dermal steady-state volumes of distribution (*V*_SS,skin_/*A*_skin_) were then calculated using Eq. 3, and the experimental dermal clearances were estimated from Eq. 6. Table 9 summarises the results and compares these *in vitro* experimental values to the *in vivo* experimental and predicted values.

**Table 9:** Measured and deduced dermato-pharmacokinetic parameters for three transdermal drugs, including the skin clearances assessed experimentally in pig skin and empirically predicted (Eq. 6)

Drug		Buprenorphine	Diclofenac^a^	Nicotine
Drug amount (μg cm^−2^)^b^				
Uptake (BUP 72 h, DF 6 h, NIC 2 h)	SC	29.2 ± 13.9	25.8 ± 3.1	7.2 ± 2.8
	Remaining skin	47.7 ± 25.9	6.8 ± 1.0	14.2 ± 5.7
	Total skin	76.8 ± 39.4	32.6 ± 3.0	21.3 ± 3.7
Clearance 1 (BUP 24 h, DF 5 h, NIC 1.5 h)	SC	25.0 ± 8.4	22.8 ± 4.8	2.8 ± 1.6
	Remaining skin	24.3 ± 12.7	7.8 ± 2.6	6.2 ± 3.0
	Total skin	49.3 ± 20.2	30.5 ± 7.0	8.9 ± 3.0
Clearance 2 (DF 17 h, NIC 3 h)	SC		25.2 ± 3.9	1.6 ± 0.7
	Remaining skin		2.1 ± 1.1	5.0 ± 2.3
	Total skin		27.3 ± 3.6	6.6 ± 3.0
Clearance 3 (DF 24 h)	SC		27.3 ± 3.5	
	Remaining skin		2.1 ± 1.2	
	Total skin		29.4 ± 3.5	
*k*_e,skin_ (h^−1^)^c^		0.016	0.005	0.42
*V*_SS_/BW (L kg^−1^)		6.1^d^	0.9^e^	2.5^d^
*K*_(skin/p)_		3.5	2.3	1.4
*V*_SS,skin_/*A*_skin_ (cm)^f^		0.50	0.33	0.21
Cl_skin_/*A*_skin_ (cm h^−1^)	*In vitro* experiment^g^	0.0080	0.0017	0.0874
	*In vivo* experiment^h^	0.0126	NA	0.0447
	predicted ^i^	0.0093	0.0086	0.0251

^a^Physicochemical properties of diclofenac: MW = 411.32 Da (diclofenac epolamine), log *P* = 4.16 (diclofenac acid), TPSA = 49.33 Å^2^, *F*_i_ = 1, *f*_u,p_ = 0.99, p*K*_a_ = 4

^b^Drug amounts (A, mean ± standard deviation, *n* = 6) recovered after uptake or clearance from the SC, epidermis/dermis, or the sum of both as indicated

^c^*k*_e,skin_ is the first-order elimination rate constant describing drug clearance from the ‘skin compartment’ in the *in vitro* experiments (see Fig. 4)

^d^Values derived from *in vivo* studies after IV administration of the drug (the values were collected from drug approval packages for the product name listed on the FDA website; see Table S1 in the Supplementary information)

^e^Value derived from *in vivo* studies after oral administration of diclofenac epolamine [37]

^f^Calculated using Eq. 3

^g^Calculated using *V*_SS,skin_/*A*_skin_ (from Eq. 3) and the experimentally (*in vitro*) determined *k*_e,skin_

^h^Calculated using *V*_SS,skin_/*A*_skin_ (from Eq. 3) and the reported *in vivo* *k*_e,terminal_ (Table 6)

^i^Predicted by Eq. 6

Although the experimental skin clearances were derived from *in vitro* studies using skin from pigs and without a functioning dermal microcirculation, the agreement between these values and those predicted by the empirical model is within a factor of 1.2 for BUP, 5 for DF and 0.29 for NIC. Given the inherent variability in the clinical and laboratory data used to derive the predicted and experimental skin clearances, the degree of overlap between measured and estimated parameters is reasonable. It is worth noting that the ‘experimental’ clearance values depend on two components: firstly, *k*_e,skin_, which is derived either from previously published *in vivo* experiments in human volunteers wearing transdermal patches, or from the *in vitro* experiments reported in this study; and, secondly, *V*_SS,skin_/*A*_skin_, which is calculated based on extrapolation of a correlation derived from experiments performed in rats. At present, whether the calculated *V*_SS,skin_/*A*_skin_ values are accurate representations of actual values in man is unknown and it is therefore impossible to say, with any confidence, how well the calculated (or predicted) values of Cl_skin_/*A*_skin_ represent human skin. What can be said and, we submit, is a key outcome of this work, is that a scheme for calculation of Cl_skin_/*A*_skin_ is now available, the robustness of which will be testable once skin-plasma drug distribution data are available for man. Finally, it should also be mentioned that this work has also demonstrated consistency between results acquired *in vitro* using pig skin data and *in vivo* in human subjects.

## Conclusions

The development of an empirical model describing drug clearance from the skin, in terms of the readily available (measured or predicted) parameters, MW, log *P* and TPSA, has been presented. The mechanistic significance of these metrics is consistent with the anticipated role of molecular size, hydrophobicity and polarity in the determination of passive drug diffusion in the skin and the compound’s eventual uptake into the systemic circulation. An attempt to validate the model’s predictive ability against (*in vitro*) experimentally derived skin clearance values of three drugs resulted in reasonable agreement.

## Supplementary Information

Below is the link to the electronic supplementary material.

Supplementary file1 (DOCX 63 KB)
