# Supplementary material for: Predicting topical drug clearance from the skin
Source: Drug Deliv Transl Res. 2020 Nov 8;11(2):729–40. doi: 10.1007/s13346-020-00864-8 (PMC7987642; doi:10.1007/s13346-020-00864-8)
Supplement: Supplementary file 1 — Supplementary file1 (DOCX 63 KB) [file 13346_2020_864_MOESM1_ESM.docx]

Supplementary Information

**Table S1.** Values of the systemic volume of distribution per body weight (V_SS_/BW) obtained from clinical studies.

| Drug | V_SS_/BW (L/kg)^a^ | Reference |
| --- | --- | --- |
| Buprenorphine | 6.1 | Butrans^®^ |
| Clonidine | 2.9 | Catapress-TTS^®^ |
| Estradiol | 1.0 | Progynova^®^ TS 100 ^b^ |
| Ethinyl estradiol | 5.0 | Akizza^® b^ |
| Fentanyl | 6.0 | Duragesic^®^ |
| Glyceryl Trinitrate | 3.3 | Nitrostat^®^ |
| Granisetron | 3.0 | [1] |
| Levonorgestrel | 1.8 | Violite^® b^ |
| Lidocaine | 1.5 | Lidoderm^®^ |
| Methylphenidate | 2.6 | Ritalin^®^ |
| Nicotine | 2.5 | Nicorette Invisi^®^ 10 mg |
| Norelgestromin | 3.0 | Ortho Evra^®^ |
| Norethisterone Acetate | 4.0 | FemHRT^®^ |
| Oxybutynin | 2.8 | Oxytrol^®^ |
| Rivastigmine | 2.2 | Exilon^®^ |
| Rotigotine | 53.8 | [2] |
| Scopolamine | 1.0 | [3] |
| Selegiline | 26.5 | [4] |
| Testosterone | 1.0 | [5] |

^a^ Information from drug approval packages for the product name listed on the FDA website (FDA-Clinical Pharmacology and Biopharmaceutics Review(s), https://www.accessdata.fda.gov/scripts/cder/daf/). When the information was not available on the FDA website, the source of V_SS_/BW is specified.

^b^ Information from the Summary of Product Characteristics (SPC) approved by the MHRA (Medicines Healthcare products Regulatory Agency, United Kingdom, https://products.mhra.gov.uk/).

**Table S2.** Values of systemic half-life and k_terminal_ calculated after removal of the patch.

| Drug | Half-life (h) (Mean ± SD)^a^ | k_terminal_ (h^-1^) | Half-life (h) | Reference |
| --- | --- | --- | --- | --- |
| Buprenorphine | 28.4 ± 5.2 | 0.025 ± 0.005 | 25.4 | ^b^Butrans^®^ |
|  |  |  | 25.3, 27.4, 32.6, 34.5, 36.8 | [6] |
|  |  |  | 32 | [7] |
|  |  |  | 25.3, 27.4 | [8] |
|  |  |  | 25.3, 27.4 | ^b^Transtec^®^ |
|  |  |  | 17.3, 26.2, 34.6 | [9] |
| Clonidine | 18.7 ± 2.2 | 0.037 ± 0.005 | 16.2, 20.4, 19.5 | [10] |
| Estradiol | 3.3 ± 1.0 | 0.219 ± 0.065 | 4.0 | [11] |
|  |  |  | 2.6 | [12] |
| Ethinyl estradiol | 17.4 ± 1.8 | 0.040 ± 0.004 | 15.0 | [13] |
|  |  |  | 13.7 | [14] |
|  |  |  | 19.9, 21 | [15] |
|  |  |  | 16.1, 16.4, 18.1, 17.1 | [16] |
|  |  |  | 15, 15.2, 17.6 | [17] |
|  |  |  | 18.1, 17.5 | [18] |
|  |  |  | 20.2 | [19] |
|  |  |  | 17.6, 18.2, 17.9 | [20] |
|  |  |  | 18.9, 17.7, 17.4 | ^b^Ortho Evra^®^ |
| Fentanyl | 21.1 ± 3.9 | 0.034 ± 0.005 | 17.0 | [21] |
|  |  |  | 21.2, 30.5 | [22] |
|  |  |  | 20.9, 20.3 | [23] |
|  |  |  | 20.6, 22.7 | [24] |
|  |  |  | 16.1 | [25] |
|  |  |  | 21.0 | [26] |
|  |  |  | 21.0 | [27] |
| Glyceryl Trinitrate | 0.6 ± 0.6 | 2.08 ± 1.96 | 0.2 | [28] |
|  |  |  | 1.0 | ^b^Nitro-DUR^®^ |
| Granisetron | 32.6 ± 2.9 | 0.021 ± 0.002 | 35.9, 30.9, 30.9 | [29] |
| Levonorgestrel | 32.9 ± 0.5 | 0.021 ± 0.000 | 32.5, 33.4 | [30] |
|  |  |  | 32.5, 32.6, 33.4 | [20] |
| Lidocaine | 6.0 ± 0.8 | 0.118 ± 0.013 | 5.2, 5.5, 5.8, 5.6 | [31] |
|  |  |  | 6.8, 7.9 | [32] |
|  |  |  | 5.6, 5.4, 6.0 | ^b^Lidoderm^®^ |
|  |  |  | 5.9, 5.8 | [33] |
| Methylphenidate | 3.5 ± 0.4 | 0.200 ± 0.023 | 4.1, 3.8, 3.6, 3.5 | [34] |
|  |  |  | 3.1, 3.0, 3.7, 3.8 | [35] |
|  |  |  | 3.0 | [36] |
| Nicotine | 3.5 ± 1.0 | 0.214 ± 0.059 | 4.1 | [37] |
|  |  |  | 4.9, 3.9, 3.7 | [38] |
|  |  |  | 4.3 | [39] |
|  |  |  | 3.2, 5.2, 3.4 | [40] |
|  |  |  | 3.9, 3.6 | [41] |
|  |  |  | 2.3, 2.4, 2.6, 2.4, 2.6, 2.5 | [42] |
|  |  |  | 5.0 | [43] |
|  |  |  | 5.6 | [44] |
|  |  |  | 2.6, 2.7 | [45] |
| Norelgestromin | 27.9 ± 3.0 | 0.025 ± 0.003 | 28.5, 32.1 | [15] |
|  |  |  | 28.2 | [13] |
|  |  |  | 25.8 | [14] |
|  |  |  | 27.6, 26.1, 28.0, 30.1 | [16] |
|  |  |  | 24.9, 28.4 | [17] |
|  |  |  | 25.9, 20.7 | [18] |
|  |  |  | 30.8, 32.9, 28.3 | ^b^Ortho Evra^®^ |
| Norethisterone Acetate | 15.0 | 0.046 | 15.0 | ^b^Evorel Conti^®^ |
| Oxybutynin | 12.3 ± 3.2 | 0.061 ± 0.020 | 7.0, 8.0 | ^b^Oxytrol^®^ |
|  |  |  | 8.0 | [46] |
|  |  |  | 13.5, 12.9 | [47] |
|  |  |  | 14.1, 15.0, 16.4 | [48] |
|  |  |  | 14.1, 13.3, 13.3 | [49] |
| Rivastigmine | 2.9 ± 0.6 | 0.248 ± 0.051 | 3.0 | [50] |
|  |  |  | 3.2, 3.3, 3.4, 4.0, 3.3 | [51] |
|  |  |  | 3.4 | [52] |
|  |  |  | 2.9, 2.7, 2.1, 2.2, 2.2, 2.1, 2.9, 2.8 | [53] |
| Rotigotine | 5.6 ± 0.6 | 0.125 ± 0.012 | 5.4 | [54] |
|  |  |  | 5.1 | [55] |
|  |  |  | 7.0 | [56] |
|  |  |  | 5.8, 5.6, 5.5, 5.1 | [2] |
|  |  |  | 5.2 | [57] |
| Scopolamine | 9.5 | 0.073 | 9.5 | ^b^Transderm Scop^®^ |
| Selegiline | 20.4 ± 0.4 | 0.034 ± 0.001 | 20.1 | [58] |
|  |  |  | 20.6 | [59] |
| Testosterone | 2.2 ± 1.0 | 0.374 ± 0.157 | 1.3 | [60] |
|  |  |  | 3.4, 2.5, 4.0 | [61] |
|  |  |  | 2.1 | [62] |
|  |  |  | 1.9 | [63] |
|  |  |  | 1.3 | [64] |
|  |  |  | 1.2 | ^b^Androderm^®^ |

^a^ Arithmetic mean ± standard deviation; ^b^ The information was collected from drug approval packages on the FDA website (FDA-Clinical Pharmacology and Biopharmaceutics Review(s), https://www.accessdata.fda.gov/scripts/cder/daf/).

**References**

1. Allen A, Davie C, Pierce D, Davy M, Upward J, Crome P et al. The pharmacokinetics of granisetron, a 5-HT3 antagonist in healthy young and elderly volunteers. European Journal of Clinical Pharmacology. 1995;48(6):519-20.

2. Cawello W, Kim SR, Braun M, Elshoff J-P, Ikeda J, Funaki T. Pharmacokinetics, safety and tolerability of rotigotine transdermal patch in healthy Japanese and Caucasian subjects. Clinical Drug Investigation. 2014;34(2):95-105.

3. Nachum Z, Shupak A, Gordon CR. Transdermal scopolamine for prevention of motion sickness. Clinical Pharmacokinetics. 2006;45(6):543-66.

4. Mahmood I. Clinical pharmacokinetics and pharmacodynamics of selegiline. Clinical Pharmacokinetics. 1997;33(2):91-102.

5. Lombardo F, Obach RS, Shalaeva MY, Gao F. Prediction of human volume of distribution values for neutral and basic drugs. 2. Extended data set and leave-class-out statistics. Journal of Medicinal Chemistry. 2004;47(5):1242-50.

6. Evans HC, Easthope SE. Transdermal buprenorphine. Drugs. 2003;63(19):1999-2010.

7. Kapil RP, Cipriano A, Friedman K, Michels G, Shet MS, Colucci SV et al. Once-weekly transdermal buprenorphine application results in sustained and consistent steady-state plasma levels. Journal of Pain and Symptom Management. 2013;46(1):65-75.

8. Kress HG. Clinical update on the pharmacology, efficacy and safety of transdermal buprenorphine. European Journal of Pain. 2009;13(3):219-30.

9. Wang Y, Cipriano A, Munera C, Harris SC. Dose‐Dependent Flux of Buprenorphine Following Transdermal Administration in Healthy Subjects. The Journal of Clinical Pharmacology. 2016;56(10):1263-71.

10. MacGregor TR, Matzek KM, Keirns JJ, van Wayjen RG, van den Ende A, van Tol RG. Pharmacokinetics of transdermally delivered clonidine. Clinical Pharmacology & Therapeutics. 1985;38(3):278-84.

11. Boyd RA, Yang BB, Abel RB, Eldon MA, Sedman AJ, Forgue ST. Pharmacokinetics of a 7‐Day 17β‐Estradiol Transdermal Delivery System: Effect of Application Site and Repeated Applications on Serum Concentrations of Estradiol and Estrone. The Journal of Clinical Pharmacology. 1996;36(11):998-1005.

12. Ginsburg ES, Gao X, Shea BF, Barbieri RL. Half-life of estradiol in postmenopausal women. Gynecologic and Obstetric Investigation. 1998;45(1):45-8.

13. Abrams LS, Skee M, Donna M, Natarajan J, Wong FA, Leese PT et al. Pharmacokinetics of norelgestromin and ethinyl estradiol delivered by a contraceptive patch (Ortho Evra™/Evra™) under conditions of heat, humidity, and exercise. The Journal of Clinical Pharmacology. 2001;41(12):1301-9.

14. Abrams LS, Skee MDM, Wong FA, Anderson MNJ, Leese PT. Pharmacokinetics of norelgestromin and ethinyl estradiol from two consecutive contraceptive patches. The Journal of Clinical Pharmacology. 2001;41(11):1232-7.

15. Abrams LS, Skee DM, Natarajan J, Wong FA, Lasseter KC. Multiple-dose pharmacokinetics of a contraceptive patch in healthy women participants☆. Contraception. 2001;64(5):287-94.

16. Abrams LS, Skee DM, Natarajan J, Wong FA, Anderson GD. Pharmacokinetics of a contraceptive patch (Evra™/Ortho Evra™) containing norelgestromin and ethinyloestradiol at four application sites. British Journal of Clinical Pharmacology. 2002;53(2):141-6.

17. Abrams LS, Skee D, Natarajan J, Wong FA. Pharmacokinetic overview of Ortho Evra™/Evra™. Fertility and Sterility. 2002;77:3-12.

18. Devineni D, Skee D, Vaccaro N, Massarella J, Janssens L, LaGuardia KD et al. Pharmacokinetics and pharmacodynamics of a transdermal contraceptive patch and an oral contraceptive. The Journal of Clinical Pharmacology. 2007;47(4):497-509.

19. van den Heuvel MW, van Bragt AJM, Alnabawy AKM, Kaptein MCJ. Comparison of ethinylestradiol pharmacokinetics in three hormonal contraceptive formulations: the vaginal ring, the transdermal patch and an oral contraceptive. Contraception. 2005;72(3):168-74.

20. Stanczyk FZ, Archer DF, Rubin A, Foegh M. Therapeutically equivalent pharmacokinetic profile across three application sites for AG200-15, a novel low-estrogen dose contraceptive patch. Contraception. 2013;87(6):744-9.

21. Varvel J, Shafer S, Hwang S, Coen P, Stanski D. Absorption characteristics of transdermally administered fentanyl. Anesthesiology. 1989;70(6):928-34.

22. Thompson J, Bower S, Liddle A, Rowbotham D. Perioperative pharmacokinetics of transdermal fentanyl in elderly and young adult patients. British Journal of Anaesthesia. 1998;81(2):152-4.

23. Marier JF, Lor M, Morin J, Roux L, Di Marco M, Morelli G et al. Comparative bioequivalence study between a novel matrix transdermal delivery system of fentanyl and a commercially available reservoir formulation. British Journal of Clinical Pharmacology. 2007;63(1):121-4.

24. Kress HG, Boss H, Delvin T, Lahu G, Lophaven S, Marx M et al. Transdermal fentanyl matrix patches Matrifen® and Durogesic® DTrans® are bioequivalent. European Journal of Pharmaceutics and Biopharmaceutics. 2010;75(2):225-31.

25. Gourlay GK, Kowalski SR, Plummer JL, Cherry DA, Gaukroger P, Cousins MJ. The transdermal administration of fentanyl in the treatment of postoperative pain: pharmacokinetics and pharmacodynamic effects. Pain. 1989;37(2):193-202.

26. Holley F, Van Steennis C. Postoperative analgesia with fentanyl: pharmacokinetics and pharmacodynamics of constant-rate iv and transdermal delivery. British Journal of Anaesthesia. 1988;60(6):608-13.

27. Portenoy RK, Southam MA, Gupta SK, Lapin J, Layman M, Inturrisi CE et al. Transdermal fentanyl for cancer pain. Repeated dose pharmacokinetics. Anesthesiology. 1993;78(1):36-43.

28. Sun JX, Piraino AJ, Morgan JM, Joshi JC, Cipriano A, Chan K et al. Comparative Pharmacokinetics and Bioavailability of Nitroglycerin and its Metabolites from Transderm‐Nitro, Nitrodisc, and Nitro‐Dur II Systems Using a Stable‐Isotope Technique. The Journal of Clinical Pharmacology. 1995;35(4):390-7.

29. Howell J, Smeets J, Drenth H-J, Gill D. Pharmacokinetics of a granisetron transdermal system for the treatment of chemotherapy-induced nausea and vomiting. Journal of Oncology Pharmacy Practice. 2009;15(4):223-31.

30. Stanczyk FZ, Rubin A, Flood L, Foegh M. Pharmacokinetics, tolerability and cycle control of three transdermal contraceptive delivery systems containing different doses of ethinylestradiol and levonorgestrel. Hormone Molecular Biology and Clinical Investigation. 2011;6(2):231-40.

31. Campbell BJ, Rowbotham M, Davies PS, Jacob III P, Benowitz NL. Systemic absorption of topical lidocaine in normal volunteers, patients with post‐herpetic neuralgia, and patients with acute herpes zoster. Journal of Pharmaceutical Sciences. 2002;91(5):1343-50.

32. Gammaitoni AR, Alvarez NA, Galer BS. Pharmacokinetics and safety of continuously applied lidocaine patches 5%. American Journal of Health-System Pharmacy. 2002;59(22):2215-20.

33. Kondamudi PK, Tirumalasetty PP, Malayandi R, Mutalik S, Pillai R. Lidocaine transdermal patch: Pharmacokinetic modeling and in Vitro–In vivo correlation (IVIVC). AAPS PharmSciTech. 2016;17(3):588-96.

34. Pierce D, Dixon CM, Wigal SB, McGough JJ. Pharmacokinetics of methylphenidate transdermal system (MTS): results from a laboratory classroom study. Journal of Child and Adolescent Psychopharmacology. 2008;18(4):355-64.

35. Patrick KS, Straughn AB, Perkins JS, González MA. Evolution of stimulants to treat ADHD: transdermal methylphenidate. Human Psychopharmacology: Clinical and Experimental. 2009;24(1):1-17.

36. Anderson VR, Scott LJ. Methylphenidate transdermal system. Drugs. 2006;66(8):1117-26.

37. Chan KK, Ross HD, Berner B, Piraino AJ, John VA. Pharmacokinetics of a single transdermal dose of nicotine in healthy smokers. Journal of Controlled Release. 1990;14(2):145-51.

38. Bannon Y, Corish J, Corrigan O, Devane J, Kavanagh M, Mulligan S. Transdermal delivery of nicotine in normal human volunteers: a single dose and multiple dose study. European Journal of Clinical Pharmacology. 1989;37(3):285-90.

39. Benowitz NL, Chan K, Denaro CP, Jacob P. Stable isotope method for studying transdermal drug absorption: the nicotine patch. Clinical Pharmacology & Therapeutics. 1991;50(3):286-93.

40. Gore AV, Chien YW. The nicotine transdermal system. Clinics in Dermatology. 1998;16(5):599-615.

41. DeVeaugh-Geiss AM, Chen LH, Kotler ML, Ramsay LR, Durcan MJ. Pharmacokinetic comparison of two nicotine transdermal systems, a 21-mg/24-hour patch and a 25-mg/16-hour patch: a randomized, open-label, single-dose, two-way crossover study in adult smokers. Clinical Therapeutics. 2010;32(6):1140-8.

42. Lin S, Ho H, Chien YW. Development of a new nicotine transdermal delivery system: in vitro kinetics studies and clinical pharmacokinetic evaluations in two ethnic groups. Journal of Controlled Release. 1993;26(3):175-93.

43. Kochak GM, Sun JX, Choi RL, Piraino AJ. Pharmacokinetic disposition of multiple-dose transdermal nicotine in healthy adult smokers. Pharmaceutical Research. 1992;9(11):1451-5.

44. Mulligan SC, Masterson JG, Devane JG, Kelly JG. Clinical and pharmacokinetic properties of a transdermal nicotine patch. Clinical Pharmacology & Therapeutics. 1990;47(3):331-7.

45. Rasmussen S, Horkan KH, Kotler M. Pharmacokinetic evaluation of two nicotine patches in smokers. Clinical Pharmacology in Drug Development. 2018.

46. Staskin DR. Transdermal systems for overactive bladder: principles and practice. Reviews in Urology. 2003;5(Suppl 8):S26.

47. Mizushima H, Takanaka K, Abe K, Fukazawa I, Ishizuka H. Stereoselective pharmacokinetics of oxybutynin and N-desethyloxybutynin in vitro and in vivo. Xenobiotica. 2007;37(1):59-73.

48. Starkman JS, Dmochowski RR. Management of overactive bladder with transdermal oxybutynin. Reviews in Urology. 2006;8(3):93.

49. Zobrist RH, Quan D, Thomas HM, Stanworth S, Sanders SW. Pharmacokinetics and metabolism of transdermal oxybutynin: in vitro and in vivo performance of a novel delivery system. Pharmaceutical Research. 2003;20(1):103-9.

50. Lefèvre G, Pommier F, Sędek G, Allison M, Huang HLA, Kiese B et al. Pharmacokinetics and bioavailability of the novel rivastigmine transdermal patch versus rivastigmine oral solution in healthy elderly subjects. The Journal of Clinical Pharmacology. 2008;48(2):246-52.

51. Lefèvre G, Sędek G, Huang HLA, Saltzman M, Rosenberg M, Kiese B et al. Pharmacokinetics of a rivastigmine transdermal patch formulation in healthy volunteers: relative effects of body site application. The Journal of Clinical Pharmacology. 2007;47(4):471-8.

52. Lefèvre G, Sędek G, Jhee S, Leibowitz M, Huang HL, Enz A et al. Pharmacokinetics and pharmacodynamics of the novel daily rivastigmine transdermal patch compared with twice‐daily capsules in Alzheimer's disease patients. Clinical Pharmacology & Therapeutics. 2008;83(1):106-14.

53. Lefèvre G, Büche M, Sedek G, Maton S, Enz A, Lorch U et al. Similar rivastigmine pharmacokinetics and pharmacodynamics in Japanese and white healthy participants following the application of novel rivastigmine patch. The Journal of Clinical Pharmacology. 2009;49(4):430-43.

54. Cawello W, Braun M, Boekens H. Absorption, disposition, metabolic fate, and elimination of the dopamine agonist rotigotine in man: administration by intravenous infusion or transdermal delivery. Drug Metabolism and Disposition. 2009;37(10):2055-60.

55. Braun M, Cawello W, Boekens H, Horstmann R. Influence of domperidone on pharmacokinetics, safety and tolerability of the dopamine agonist rotigotine. British Journal of Clinical Pharmacology. 2009;67(2):209-15.

56. Cawello W, Ahrweiler S, Sulowicz W, Szymczakiewicz‐Multanowska A, Braun M. Single dose pharmacokinetics of the transdermal rotigotine patch in patients with impaired renal function. British Journal of Clinical Pharmacology. 2012;73(1):46-54.

57. Cawello W, Kim SR, Braun M, Elshoff J-P, Masahiro T, Ikeda J et al. Pharmacokinetics, safety, and tolerability of rotigotine transdermal system in healthy Japanese and Caucasian subjects following multiple-dose administration. European Journal of Drug Metabolism and Pharmacokinetics. 2016;41(4):353-62.

58. Azzaro AJ, Ziemniak J, Kemper E, Campbell BJ, VanDenBerg C. Pharmacokinetics and absolute bioavailability of selegiline following treatment of healthy subjects with the selegiline transdermal system (6 mg/24 h): a comparison with oral selegiline capsules. The Journal of Clinical Pharmacology. 2007;47(10):1256-67.

59. Rohatagi S, Barrett JS, Dewitt KE, Morales RJ. Integrated pharmacokinetic and metabolic modeling of selegiline and metabolites after transdermal administration. Biopharmaceutics & Drug Disposition. 1997;18(7):567-84.

60. Raynaud J-P, Aumonier C, Gualano V, Betea D, Beckers A. Pharmacokinetic study of a new testosterone-in-adhesive matrix patch applied every 2 days to hypogonadal men. The Journal of Steroid Biochemistry and Molecular Biology. 2008;109(1-2):177-84.

61. Findlay JC, Place VA, Snyder PJ. Transdermal delivery of testosterone. The Journal of Clinical Endocrinology & Metabolism. 1987;64(2):266-8.

62. Singh AB, Norris K, Modi N, Sinha-Hikim I, Shen R, Davidson T et al. Pharmacokinetics of a transdermal testosterone system in men with end stage renal disease receiving maintenance hemodialysis and healthy hypogonadal men. The Journal of Clinical Endocrinology & Metabolism. 2001;86(6):2437-45.

63. Meikle AW, Mazer NA, Moellmer JF, Stringham JD, Tolman KG, Sanders SW et al. Enhanced transdermal delivery of testosterone across nonscrotal skin produces physiological concentrations of testosterone and its metabolites in hypogonadal men. The Journal of Clinical Endocrinology & Metabolism. 1992;74(3):623-8.

64. Meikle AW, Arver S, Dobs AS, Sanders SW, Rajaram L, Mazer NA. Pharmacokinetics and metabolism of a permeation-enhanced testosterone transdermal system in hypogonadal men: influence of application site--a clinical research center study. The Journal of Clinical Endocrinology & Metabolism. 1996;81(5):1832-40.
